# Evolutionary Mechanisms of Microbial Genomes 2012

**DOI:** 10.1155/2012/872768

**Published:** 2012-08-22

**Authors:** Hiromi Nishida, Shinji Kondo, Hideaki Nojiri, Ken-ichi Noma, Kenro Oshima

**Affiliations:** ^1^Agricultural Bioinformatics Research Unit, Graduate School of Agricultural and Life Sciences, The University of Tokyo, Tokyo 113-8657, Japan; ^2^Laboratory for Cellular Systems Modeling, RIKEN Research Center for Allergy and Immunology, Kanagawa 230-0045, Japan; ^3^Laboratory of Environmental Biochemistry, Biotechnology Research Center, The University of Tokyo, Tokyo 113-8657, Japan; ^4^Gene Expression and Regulation Program, The Wistar Institute, 3601 Spruce Street, Room 116, Philadelphia, PA 19104, USA; ^5^Department of Agricultural and Environmental Biology, Graduate School of Agricultural and Life Sciences, The University of Tokyo, Tokyo 113-8657, Japan

What is the driving force in the course of microbial genome evolution? What is the mechanism for distinguishing self-genome from others? These fundamental questions remain elusive although rigorous studies are underway by using comparative genomics. The special issue “*Evolutionary mechanisms of microbial genomes*” has been launched in 2011 and presented 11 original papers. Here, this new version in 2012 presents 10 papers (one review and nine research articles).

Two papers are presented in phylogenomics. K. Oshima et al. revealed a close relationship of Aquificales to Thermotogales based on the whole-genome comparison in “*Phylogenetic position of Aquificales based on the whole genome sequences of six Aquificales species*.” An extensive and elaborate review of fish pathogenic bacteria has been presented by P. S. Sudheesh et al. in “*Comparative pathogenomics of bacteria causing infectious diseases in fish*.”

Two papers are presented on subjects related to evolution of base composition in genomes. H. Nishida et al. in “*Genome signature difference between Deinococcus radiodurans and Thermus thermophilus*” observed distinct tetranucleotide frequencies between the genomes of *D. radiodurans* and *T. thermophilus*, potentially reflecting different evolutionary backgrounds of the two species after divergence from common ancestor. H. Nishida in “*Comparative analyses of base compositions, DNA sizes, and dinucleotide frequency profiles in archaeal and bacterial chromosomes and plasmids*” reported lower GC content (by up to ~10%) of plasmids compared to their host chromosomes and higher correlation of GC content and chromosome size in bacteria than in archaea.

Two papers are presented about horizontal gene transfer in genome evolution. M. Jalasvuori in “*Vehicles, replicators, and intercellular movement of genetic information: Evolutionary dissection of a bacterial cell*” discussed a hypothesis that any given biosphere comprising prokaryotic cell vehicles and genetic replicators may naturally evolve toward possessing horizontally moving replicators of various types. V. S. Pylro et al. described horizontal gene transfer events of the gene *dszC* involved in the cleavage of carbon-sulfur bonds in “*Detection of horizontal gene transfers from phylogenetic comparisons*.”

An article about DNA mutation is presented by Y. Shiwa et al. in “*Whole-genome profiling of a novel mutagenesis technique using proofreading-deficient DNA polymerase *δ**.” They compared mutations created by the chemical mutagen ethyl methanesulfonate (EMS) and the proofreading-deficient DNA polymerase *δ* and found that the mutations created by the proofreading-deficient DNA polymerase *δ* generated more diverse amino acid substitution patterns than those by EMS.

Three papers are presented on subjects related to metabolic pathway. H. Nishida in “*Comparative analyses of homocitrate synthase genes of ascomycetous yeasts*” described gene duplications of the homocitrate synthase which have occurred multiple times during evolution of the ascomycetous yeasts. H. Nishida and M. Nishiyama in “*Evolution of lysine biosynthesis in the phylum Deinococcus-Thermus*” reported that bacterial lysine biosynthesis genes of the common ancestor of the *Deinococcus-Thermus* phylum used the *α*-aminoadipate pathway instead of the diaminopimelate pathway. K. Ueda et al. in “*Dispensabilities of carbonic anhydrase in Proteobacteria*” analyzed the distribution of carbonic anhydrase (CA) in proteobacteria, compared CA-retaining and CA-deficient genomes, and found absence of coding sequence in some strains and frame shifts in others.

In closing this introduction to the special issue, we would like to express our full appreciation to all the authors and reviewers for their enormous efforts that have made the timely completion of our assignment successful. We sincerely hope that this special issue will stimulate further the investigation of evolutionary mechanisms of microbial genomes.

*Hiromi Nishida* *Hiromi Nishida* *Shinji Kondo* *Shinji Kondo* *Hideaki Nojiri* *Hideaki Nojiri* *Ken-ichi Noma* *Ken-ichi Noma* *Kenro Oshima* *Kenro Oshima*

